# Acute Psychosis Due to Anti-N-Methyl D-Aspartate Receptor Encephalitis Following COVID-19 Vaccination: A Case Report

**DOI:** 10.3389/fneur.2021.764197

**Published:** 2021-11-04

**Authors:** Patrick Flannery, Ingrid Yang, Madjid Keyvani, George Sakoulas

**Affiliations:** ^1^The Salk Institute of Biological Studies, San Diego, CA, United States; ^2^Sharp Rees-Stealy Medical Group and Sharp Memorial Hospital, San Diego, CA, United States; ^3^Division of Host-Microbe Systems and Therapeutics, Center for Immunity, Infection and Inflammation, University of California-San Diego School of Medicine, La Jolla, CA, United States

**Keywords:** COVID-19, vaccine, NMDA-receptor, encephalitis, psychosis

## Abstract

Anti-N-methyl D-aspartate (NMDA) receptor (anti-NMDAR) encephalitis has been reported after SARS-CoV-2 infection, but not after SARS-CoV-2 vaccination. We report the first known case of anti-NMDAR encephalitis after SARS-CoV-2 immunization in a young female presenting with acute psychosis, highlighting a rare potential immunological complication of vaccination against SARS-CoV-2 that is currently being distributed worldwide. The patient presented initially with anxiety and hypochondriacal delusions which progressed to psychosis and catatonia but returned to baseline with aggressive immunomodulatory therapy consisting of intravenous immunoglobulin, high-dose glucocorticoids, and rituximab. This study highlights that the workup of acute psychosis should include establishing a history of recent vaccination followed by a thorough neurological assessment, including for anti-NMDAR antibodies in blood and cerebrospinal fluid.

## Introduction

Anti-N-methyl-D-aspartate receptor (anti-NMDAR) encephalitis is an autoimmune mediated condition characterized by complex neuropsychiatric syndromes and the presence of antibodies against the GluN1 receptors in the CSF (1). This disease was first described in 2007 as a paraneoplastic syndrome in women presenting with ovarian teratomas but has since been designated as the second most common immune mediated encephalopathy (2, 3). Anti-NMDAR encephalitis has been associated with viral illnesses such as Japanese encephalitis, HSV-1, Epstein-Barr virus, and most recently COVID-19 infection (Table 1) (5, 9–11). Additionally, anti-NMDAR encephalitis has been associated with vaccinations against H1N1, yellow fever, TdaP-IPV booster, and the Japanese Encephalitis (3, 12–14). In this case report, we present the first instance of anti-NMDAR encephalitis after receiving the Pfizer-BioNTech COVID-19 vaccine.

**Table 1:** Published cases reports of anti-NMDA encephalitis secondary to COVID-19 infection.

**Patient**	**Gender**	**Age**	**Outcome**	**Anti-NMDA diagnosis from onset of primary disease**	**Time from positive COVID-19 test to anti-NMDA diagnosis**	**Neurological symptoms**	**Neurological findings**
1 (4)	M	20's	Condition improving at time of print	3 weeks	3 weeks	**Initial presentation:** psychomotor agitation, disorganized speech, anxiety, persecutory delusions and auditory hallucinations, and global insomnia	**Initial presentation:** cerebral CT scan was negative for acute neuroanatomical abnormalities
2 (5)	F	<2	Return to baseline 1 month after onset of initial symptoms	2 weeks	1 week	**Initial presentation:** fever, fussiness, poor sleep, constipation, and decreased oral intake. **Week 1:** constant thrashing movements of extremities, non-communicative, and seizures. **Week 2:** worsening encephalopathy and persistent hyperkinetic movements of the arms, legs, and head.	**Week 1:** CSF analysis demonstrated a glucose of 56 mg/dL (serum 105 mg/dL), total protein 25 mg/dL, seven leukocytes/μL (89% lymphocytes and 11% monocytes), and two red blood cells/μL. **Week 2:** NMDAR-IgG positivity in the serum (1:640) and CSF (1:40).
3 (6)	M	<10	Released with mildly ataxic gait	12 days	5 days	**Initial presentation:** ataxia, wide-based gait, loss of DTR, somnolence, and seizures. **Week 2:** choreiform movements in the hands and feet, tongue protrusion, bruxism, lip smacking, agitation, catatonia, echolalia, and tachycardia. **Week 4**: focal seizure	**Initial presentation:** MRI and CSF analysis was normal awake and sleep EEGs were encephalopathic with widespread delta waves. **Week 4:** normal MRI
4 (7)	M	50's	Patient was discharged with no neurological deficits and in good condition after 4 months	8 days	−1 days	**Initial presentation:** Confabulations and delirious ideas. **Day 4:** focal motor seizures with impaired awareness and oro-facial dyskinesia/automatisms appeared. **Week 1:** refractory status epilepticus, oro-facial dyskinesias, loss of consciousness.	**Initial presentation:** 2 brain MRI's negative in the first week.
5 (8)	F	Teens	Full recovery after 2 months treatment	3.5 weeks	Same Day	**Initial presentation**: 3-week history of mood change as depression and anhedonia accompanied by lack of concentration and Generalized Tonic-Clonic seizures.	**Initial presentation: g**eneralized brain edema, minor meningismus, and neck stiffness. CSF analysis showed semi turbid, light pink fluid. WBC: 27, RBC: 1,997, lymphocytes: 93%, PMN: 7%, glucose: 55 mg/dL, and protein: 241 mg/dL

## Case Narrative

**Figure 1:**
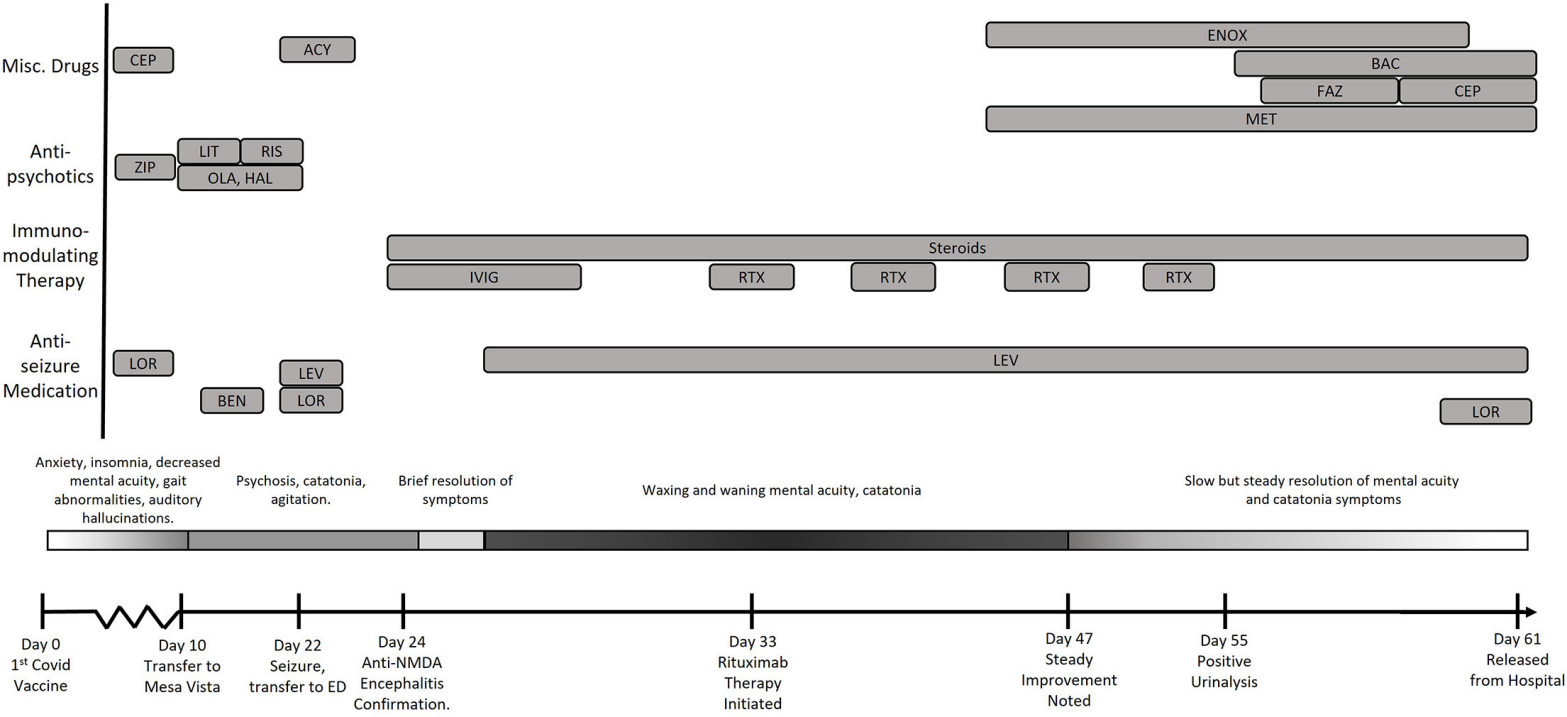
Timeline of the evolution of the patient's condition in relation to treatments and findings. CEP, Cephalexin; LEV, Levetiracetam; LOR, Lorazepam; RIX, Rituximab; FAZ, Cefazolin; ENOX, Enoxaparin; MET, Metoprolol; BAC, Bactrim; ZIP, Ziprasidone; OLA, Olanzapine; HAL, Haldol; RIS, Risperadol; Ben, Benztropine.

A female in her 20's presented to the Emergency Department (ED) with a chief complaint of urinary frequency 1 week after receiving her first dose of the Pfizer-BioNTech COVID-19 vaccine (Figure 1). The patient's family stated she had increasingly frequent bouts of anxiety, decreased mentally acuity, insomnia, and a fixation that she suffered from irritable bowels and kidney disease. She displayed waxing and waning hypochondriacal delusions that she had contracted COVID-19 and that “her body was shutting down.” The patient was also noted to have some motor dysfunction and a transient bout of aphasia during this time. There were no complaints of antecedent infection, fever, or headache. Family history and past medical history were non-contributary. The physical examination showed tachycardia and hypertension but otherwise unremarkable. Hematology and metabolic labs and urinalysis were normal.

The patient was discharged from the ED with instructions to follow up with her primary care physician but returned the following day with complaints of increasing anxiety as well as continued somatization of bowel and kidney disease. The patient also endorsed accusatory auditory hallucinations but denied suicidal or homicidal ideation. Repeat blood tests demonstrated mild leukocytosis and slightly increased alanine aminotransferase (ALT) and aspartate aminotransferase (AST). The physical exam was again normal except for elevated blood pressure and tachycardia. SARS-CoV-2 nasopharyngeal polymerase chain reaction (PCR) was negative. Due to the persistent tachycardia and hypertension, she was kept overnight for observation. No cerebrospinal fluid (CSF) analysis was performed during these initial two ED visits.

The following morning the patient removed her clothing and had a bowel movement on the floor. With no discovery of metabolic or toxic causes based on bloodwork and imaging for her acute psychosis, she was transferred to an inpatient psychiatric unit for voluntary admission. Treatment was begun with olanzapine and haloperidol (5 mg, Q4H). Despite these therapies, patient became increasingly psychotic, which was initially managed with lithium, but this was discontinued due to symptoms of catatonia. Risperidone therapy was then trialed (0.5 mg, Q4H), however the patient experienced a grand mal seizure which prompted transfer back to the ED and subsequent admission to the intensive care unit. Detailed question of family members revealed no evidence of upper respiratory, gastrointestinal, or other antecedent illness in the preceding few weeks.

At that time, the patient's memory was intact, and she was responsive to questions but lethargic. She also exhibited symptoms of ongoing catatonia, answered questions in short sentences in a monotonous tone with low phonation. She could ambulate, but slowly and with a one-person assist mainly to aid with initiation of movement. Computerized tomography (CT) and magnetic resonance imaging (MRI) of the brain were normal, as were and hematologic and metabolic laboratory evaluations. Eventually, a lumbar puncture was performed, and patient's CSF analysis showed a mild lymphocyte pleocytosis with 12–14 nucleated cells/mm^3^. CSF polymerase chain reaction studies for enterovirus, herpes simplex, varicella zoster, and Epstein-Barr virus were negative. Blood serologies for Mycoplasma pneumoniae, and HIV were negative. Studies for Cryptococcus neoformans and Coccidioides immitis were negative.

The constellation of symptoms (spontaneous defecation, catatonia, sudden encephalopathy without metabolic or infectious findings) coupled with the preliminary CSF results and the history of deterioration after SARS-CoV-2 vaccination led to a strong clinical suspicion of an autoimmune-mediated encephalitis driven by the vaccine. While awaiting for the appropriate diagnostic test results, a 5-day course of IVIG (25 g given at 0.5 mg/kg/min initially and increased to 8 mg/kg/min as tolerated) and steroid treatment (methylprednisolone, 40 mg Q8H) were begun, with clinical improvement demonstrated within 24 h. The patient become fully alert and oriented, with increased dexterity and decreased catatonia, although her affect remained flat. Additionally, metoprolol was prescribed to manage the patient's tachycardia and hypertension.

Eventually, CSF anti-NMDA titers of 1:20 returned. Due the high correlation between anti-NMDAR encephalitis and paraneoplastic teratomas, a transvaginal ultrasound, chest x-ray and CT, and MRI of the chest, pelvis, and abdomen were performed, all of which were unremarkable. EEG revealed no abnormalities. When the 5-day IVIG course was completed, her neurological status deteriorated. The patient continued to present with a flat affect and increasingly poor volitional initiation of movement and speech.

Weekly rituximab therapy was initiated (375 mg/m^2^ initial infusion of 50 mg/h increased to a maximum of 400 mg/h), and methylprednisolone dose was increased. The patient's mental acuity and catatonia continued to wax and wane in severity until the administration of her third dose of Rituximab. Repeat evaluation of CSF revealed resolution of her lymphocytic pleocytosis (4 WBC/mm^3^, normal ≤) Anti-NMDAR CSF titers had decreased to 1:10. Slow but consistent improvements in her neurological status were observed following her third rituximab dose. Eventually, 45 days in the hospital and 61 days after receiving the SARS-CoV-2 vaccine, she was discharged from the hospital with minor neurological deficits. She remains well 3 months after hospital discharge on anticonvulsant therapy, with no signs of relapse and has returned to work.

## Discussion

SARS-CoV-2 vaccines have been critical in reducing COVID-19 morbidity and mortality and facilitated the societal return to normalcy during the COVID-19 pandemic. While numerous psychiatric conditions, including anti-NMDAR encephalitis, have been shown to complicate COVID-19 infections (Table 1), this case report is the first reported incident of anti-NMDAR encephalitis temporally linked to SARS-CoV-2 vaccination (4, 5, 7, 8, 15). Extensive workup showed no evidence that patient's symptoms were due to a paraneoplastic condition, which is more common for this specific neurologic condition. Instead, based on precedent cases of anti-NMDAR encephalitis caused by vaccines against influenza, yellow fever, Japanese encephalitis, and tetanus/diphtheria vaccines, clinicians in this case quickly considered the recent receipt of SARS-CoV-2 vaccine as a possible trigger of anti-NMDAR encephalitis (3, 12–14).

The diagnosis of an auto-immune encephalitis was not considered during the initial presentation to the emergency room because the anxiety, hallucinations, insomnia, psychosis, and were initially considered symptoms of primary psychiatric diseases, further supported by absence of fever or other objective signs of systemic infection or inflammation. Therefore, one of the major teaching points of this case is the need for serious consideration of organic nervous system causes of psychosis, despite the absence of recent or current signs of infection. This requires careful imaging of the brain, performance of a lumbar puncture, and the search for anti-NMDAR in both CSF and peripheral blood. There have been two recent studies on the psychiatric manifestations of anti-NMDAR encephalitis which report that severe agitation, speech disturbance, and catatonia amongst other psychiatric features, may signal the presence of organic pathology, particularly when dealing with a young female presenting at an atypical age for primary psychosis (16). The concomitant presence of seven features-agitation, aggression, hallucinations, delusions, mutism, irritability or mood instability, and depressed mood would not be typical of any single psychiatric diagnosis and point to organic brain pathology (17).

Despite initial delay in establishing the diagnosis, the presentation of seizure, psychiatric symptoms, and history of recent of SARS-CoV-2 vaccination resulted in prompt treatment with IVIG and glucocorticoids even before the diagnosis was established *via* anti-NMDAR antibody detection. This provided temporary improvement of symptoms, perhaps due to the blockade of harmful anti-NMDAR antibodies driving the disease. However, it was not until rituximab-mediated B-lymphocyte depletion blocking formation of new anti-NMDAR antibodies that the patient's symptoms finally started to show signs of improvement (6). However, given the long 3-week half-life of IgG, the process of clinical improvement was very slow, dependent on gradual clearance of previously formed anti-NMDAR antibodies induced by the vaccine while rituximab prevented formation of new antibodies. We hypothesize that concomitant plasmapheresis alongside rituximab may have hastened the neurological recovery by more rapid removal of harmful anti-NMDAR antibodies.

In summary, we present the first case of anti-NMDAR encephalitis complicating SARS-CoV2 vaccination in a previously healthy young woman. This case provides an important reminder that (i) psychiatric clinical presentations warrant a thorough medical workup, including brain imaging, CSF analysis, anti-NMDAR antibody testing, and a vaccine history; (ii) combined therapies of blocking (IVIG), reducing production of (rituximab), and even removing (plasmapheresis) harmful anti-NMDAR may be the optimal strategy to reverse the neurological and psychiatric symptoms driven by anti-NMDAR antibody production. Fortunately, prompt therapy targeting anti-NMDAR antibodies resulted in achieving and sustaining an excellent clinical outcome. In addition to providing clinicians the opportunity to identify potential vaccine-associated anti-NMDAR encephalitis, particular attention may be needed to patients receiving COVID-19 vaccine who have previously have had anti-NMDAR encephalitis.

## Data Availability Statement

The original contributions presented in the study are included in the article/supplementary material, further inquiries can be directed to the corresponding author/s.

## Ethics Statement

Ethical review and approval was not required for the study on human participants in accordance with the local legislation and institutional requirements. Written informed consent for participation was not required for this study in accordance with the national legislation and the institutional requirements.

## Author Contributions

PF wrote the first draft of the paper and compiled background information. IY, MK, and GS were treating physicians and edited manuscript draft. All authors contributed to the article and approved the submitted version.

## Conflict of Interest

The authors declare that the research was conducted in the absence of any commercial or financial relationships that could be construed as a potential conflict of interest.

## Publisher's Note

All claims expressed in this article are solely those of the authors and do not necessarily represent those of their affiliated organizations, or those of the publisher, the editors and the reviewers. Any product that may be evaluated in this article, or claim that may be made by its manufacturer, is not guaranteed or endorsed by the publisher.
